# Brain Monoamine Dysfunction in Response to Predator Scent Stress Accompanies Stress-Susceptibility in Female Rats

**DOI:** 10.3390/biom13071055

**Published:** 2023-06-29

**Authors:** Courtney S. Wilkinson, Harrison L. Blount, Marek Schwendt, Lori A. Knackstedt

**Affiliations:** 1 Psychology Department, University of Florida, Gainesville, FL 32611, USA; harrisonblount@ufl.edu (H.L.B.); schwendt@ufl.edu (M.S.); knack@ufl.edu (L.A.K.); 2 Center for Addiction Research and Education, University of Florida, Gainesville, FL 32611, USA; 3 Center for OCD and Anxiety Related Disorders, University of Florida, Gainesville, FL 32611, USA

**Keywords:** predator scent stress, anxiety, anhedonia, serotonin, norepinephrine, dopamine

## Abstract

Post-traumatic stress disorder (PTSD) is prevalent in women; however, preclinical research on PTSD has predominantly been conducted in male animals. Using a predator scent stress (PSS) rodent model of PTSD, we sought to determine if stress-susceptible female rats show altered monoamine concentrations in brain regions associated with PTSD: the medial prefrontal cortex (mPFC), nucleus accumbens (NAc), and dorsal (dHIPP) and ventral (vHIPP) hippocampus. Female Sprague–Dawley rats were exposed to a single, 10-min PSS exposure and tested for persistent anhedonia, fear, and anxiety-like behavior over four weeks. Rats were phenotyped as stress-Susceptible based on sucrose consumption in the sucrose preference task and time spent in the open arms of the elevated plus maze. Brain tissue was collected, and norepinephrine, dopamine, serotonin, and their metabolites were quantified using high-performance liquid chromatography. Stress-susceptibility in female rats was associated with increased dopamine and serotonin turnover in the mPFC. Susceptibility was also associated with elevated dopamine turnover in the NAc and increased norepinephrine in the vHIPP. Our findings suggest that stress-susceptibility after a single stress exposure is associated with long-term effects on monoamine function in female rats. These data suggest interventions that decrease monoamine turnover, such as MAOIs, may be effective in the treatment of PTSD in women.

## 1. Introduction

Post-traumatic stress disorder (PTSD) is a psychiatric disorder characterized by anxiety, anhedonia, and vigilance that persists for months to years after a trauma exposure [1,2,3]. Only 10–25% of individuals exposed to trauma will develop PTSD, with an increased prevalence in females [4,5]. The rate of remission from PTSD symptoms five months post-trauma exposure is approximately 37%, suggesting a need for further advancements in treatment options [6]. Understanding the pathophysiology of PTSD is imperative for identifying future treatments. 

Accumulating evidence suggests the monoamine system is implicated in PTSD. Clinical Pharmacotherapies targeting serotonin show moderate efficacy in treating symptoms of PTSD, with the selective serotonin reuptake inhibitors (SSRIs) sertraline and paroxetine currently being the only medications approved by the FDA for PTSD [7]. Patients with PTSD show low peripheral (platelet) serotonin compared to healthy controls [8]. In addition to serotonin, the noradrenergic activity of these SSRIs [9,10], together with off-label use and efficacy of α- and β adrenergic blockers (such as prazosin and propranolol) for PTSD treatment [11,12,13], suggest a role of norepinephrine (NE) in this disorder. Further, elevated urinary and cerebrospinal fluid NE is observed in men with combat-related PTSD [14,15]. Dopamine (DA) is also implicated in PTSD—elevated urinary DA is observed in men and women with PTSD, as well as elevated cerebrospinal fluid NE in males [15,16]. Preclinical investigation is needed to fully understand the role of monoamines in PTSD, as clinical findings are limited to peripheral or cerebrospinal fluid readouts.

The neurobiology underlying susceptibility to developing long-term symptoms following a single stressor can be examined using preclinical models, of which there are several (e.g., single prolonged stress, early life stress, underwater stress, chronic variable stress, etc.). Preclinical models that consider susceptibility and resilience to the long-term effects of a single stress exposure particularly have face validity for the study of PTSD [17]. Two such models are the predator scent stress (PSS) and social defeat model, which assess anhedonia (e.g., sucrose preference, sucrose consumption, and forced swim), social avoidance (e.g., social interaction ratio), and/or anxiety-like behavior (e.g., elevated plus maze; EPM, acoustic startle response; ASR) days-weeks after the stressor. Next, statistical approaches (e.g., double median-split) are used to phenotype rats as stress-Susceptible or -Resilient [17,18,19,20,21,22,23]. 

Brain monoamine dysregulation underlying PTSD, anxiety, and depression has been investigated at the preclinical level exclusively in male rodents, both using models that consider susceptibility to stress and those that compare the entire population of stressed rodents to unstressed controls. Quantification of brain monoamines via ex vivo tissue punch analysis following footshock, forced swim, social defeat/isolation, predator/predator scent, and chronic/repeated stress find disrupted monoamine system function in the medial prefrontal cortex (mPFC), nucleus accumbens (NAc), and hippocampus (HIPP) associated with stress exposure [24,25,26,27,28] and stress-susceptibility [25,29,30,31]. Since PTSD is more prevalent in women and preclinical research indicates sex-specific alterations in stress susceptibility in rodents [32], examining the long-term effect of stress on brain monoamines in stress-Susceptible female rats is imperative. 

The present study aims to test the hypothesis that the persistent anxiety- and anhedonia-like behavior observed in females after PSS is associated with disrupted monoamine concentrations in key brain regions associated with PTSD: mPFC, NAc, and the dorsal (dHIPP) and ventral (vHIPP) hippocampus. We hypothesize that, as in males, stress susceptibility in females will be associated with NE and DA dysregulation, specifically increased NE in the mPFC, dHIPP, and vHIPP and increased DA, DOPAC, and DA turnover in the mPFC; increased DA in the NAc; and decreased HVA and increased DOPAC in the hippocampus. We also expect to observe decreases in the 5-HT metabolite 5-hydroxyindoleacetic acid (5-HIAA) in the mPFC, decreased 5-HT and 5-HT turnover in the mPFC, dHIPP, and vHIPP, and increased 5-HT and 5-HT turnover in the NAc of stress-Susceptible females. 

## 2. Materials and Methods

### 2.1. Animals

Fifty-four female Sprague–Dawley rats arrived at 8 weeks of age and were individually housed in ventilated cages on a reverse 12-h light/dark cycle (lights off at 7 a.m.). Rats habituated to the vivarium for 7 days before the experiment started with access to standard rat chow and water ad libitum. Following stress or control exposures, subjects were food-restricted to 20 g/day. All procedures were authorized and approved by the University of Florida’s Institutional Animal Care & Use Committee.

### 2.2. Stress Exposure 

Rats were placed into a cylindrical, plexiglass container (Bio Bubble Pets, Boca Raton, FL, USA) with mesh flooring for 10 min. Below the mesh floor was filter paper blotted with 3 µL of predator scent (2,5-dihydro-2,4,5-trimethylthiazoline; TMT, 97% purity, BioSRQ; *n* = 40) or unscented paper (Control, *n* = 14). Rats could not interact with the filter paper and new paper was used for each rat. Chambers were cleaned with 70% ethanol between rats. Exposure sessions were recorded to measure time spent freezing. Immediately after TMT/control exposure, vaginal lavage was used to collect samples to determine the estrous cycle phase (estrus, pro-estrus, or met/diestrus), as described in Blount et al. [32]. 

### 2.3. Elevated Plus Maze and Acoustic Startle Response

Anxiety-like behavior on the elevated plus maze and acoustic startle response task was assessed one week after PSS exposure. This timing of assessment of anxiety-like behavior is the standard in the predator scent stress model [17,18,19,20]. Rats were placed into the center zone of the EPM (L × W: 10 × 10 cm; Med Associates, St. Albans, VT, USA) at task start and permitted to freely roam the opposing open and closed arms (L × W × H: 51 × 10 × 40.5 cm) for 5 min. The EPM was elevated 50 cm above the floor and the test room was illuminated to 50 lux. Time spent in the open arms (OA), closed arms (CA), and the number of open and closed arm entries were quantified by Ethovision XT 14 software (Noldus Information Technology, Leesburg, VA, USA).

Immediately after the EPM test, rats received a 30-min trial of the ASR (SR-LAB^TM^ Startle System, San Diego Instruments, San Diego, CA, USA; 51 × 55 × 31 cm). ASR chambers were ventilated and equipped with two speakers, a holding tube with plexiglass partitions, and a transducer system for startle detection using Advanced Startle Software (San Diego Instruments, San Diego, CA, USA). After 5 min of habituation to 68 dB white noise, rats received 30 intermittent startle trials of 110 dB white noise separated by a 30–45 s inter-trial interval. Mean startle amplitude and percent habituation (mean of the last 6 ASR trials divided by the mean of the first 6 trials, multiplied by 100) were assessed. Immediately after the ASR test, vaginal lavage was used to collect samples to determine the estrous cycle phase. 

### 2.4. Sucrose Preference Test

Sixteen days after TMT or control exposure, rats were tested for anhedonia using a 48-hsucrose (32%) preference test [23,33,34,35]. Rats received 2-bottle choice access to either water or a 32% sucrose-water solution for 48 h in 50 mL bottles distinct from home-cage water bottles. Liquid consumption was recorded every 12 h and bottles were refilled. The placement of bottles was rotated after 24 h. Percent preference (mL of sucrose consumed divided by mL total liquid consumed, multiplied by 100) and total sucrose consumed were measured.

### 2.5. Light–Dark Box 

Twenty-one days after TMT or control exposure, rats were tested for persistent anxiety-like behavior in the light–dark (L-D) box test. Rats were placed into plexiglass chambers (L × W × H: 40 × 44 × 37 cm) with an opaque black plexiglass insert (L × W × H: 20 × 44 × 37 cm) used to darken one side of the chamber with an opening to allow movement between light and dark sides for 10 min. The illumination of the light compartment was adjusted to ~300 lux. Total time spent in the dark and light chambers and latency to enter the light and dark chambers were quantified.

### 2.6. Stress Context Re-Exposure 

**Figure 1:**
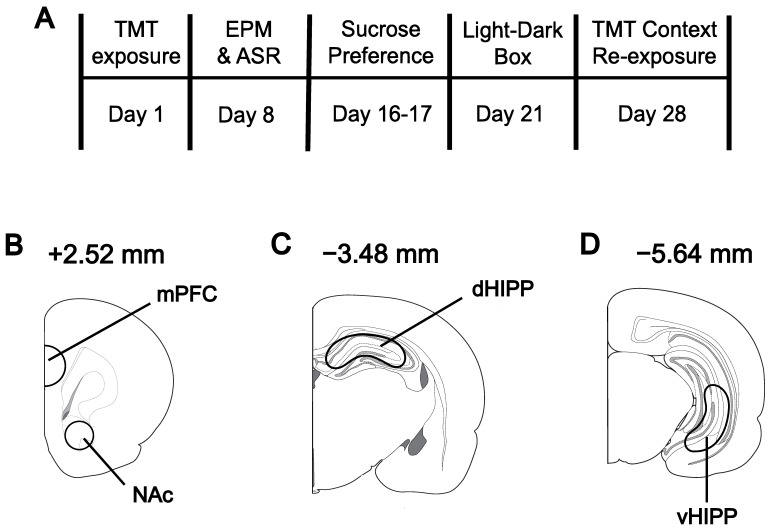
Experimental timeline (**A**) and schematic of tissue dissection areas for the medial prefrontal cortex (mPFC) and nucleus accumbens (NAc) (**B**), dorsal hippocampus (dHIPP); (**C**), and ventral hippocampus (vHIPP) (**D**). Coordinates denoted relative to bregma.

Twenty-eight days after TMT or control exposure, rats were re-exposed to the odor context for a 5-min contextual fear test. Two hours later, rats were rapidly decapitated, and brains were bilaterally dissected. This time point was selected because the neurotransmitter and metabolites of interest return to baseline after 100 min following exposure to stress or a novel environment [36,37,38,39,40,41,42,43,44]. Brains were dissected, flash-frozen in 2-methylbutane, and preserved at −80 °C. One hemisphere was used for the present study and the other was used for the previously published analysis of mGlu5 mRNA expression [32]; samples in the present study were equally distributed between the left and right hemispheres. See Figure 1 for the timeline. 

### 2.7. Tissue Collection and High-Performance Liquid Chromatography

Brain regions of interest were identified using Paxinos and Watson’s rat brain atlas [45] relative to bregma (prelimbic mPFC: 3.72 to 2.52 mm, NAc: 2.52 to 1.8 mm, dHIPP: −3.12 to −4.68 mm, vHIPP: −5.28 to −6.00 mm; Figure 1B–D) and dissected from a series of 300 mm-thick sections collected using micropunches (Harris Uni-Core, Ted Pella, Redding, CA; mPFC = 2 mm; NAc = 1.5 mm; dHIPP and vHIPP = 3.00 mm punch manually altered to fit brain region; see Figure 1C,D). Tissue was then weighed, homogenized by sonication in 200 µL of 0.2 M perchloric acid, and centrifuged at 1600× *g* for 5 min at 4 °C to remove cellular debris. The supernatant was collected using filtered pipetted tips, and 10 µL was injected into HPLC with electrochemical detection (Thermo Fisher Scientific, Waltham, MA, USA) for analysis. Samples were injected into a KROMASIL C-18 column (3.5 µm, 100A, 3.0 × 150 mm; Sigma-Aldrich, Burlington, MA, USA) at 0.4 µL/min. The mobile phase (75 mM sodium dihydrogen phosphate monohydrate, 1.7 mM 1-octanesulfonic acid sodium salt, 100 µL/L triethylamine, 25 µM ethylendiaminetetraacetic acid dipotassium salt dihydrate, and 10% acetonitrile, pH = 3) was adapted from Perrine et al. [46]. Cell potentials were set to −150 mV and +220 mV. Absolute tissue values for NE, DOPAC, DA, HVA, 5-HIAA, and 5-HT were calculated based on external standards and expressed as pg/mg of tissue.

### 2.8. Statistical Analysis 

All analyses were conducted using PRISM (v.9.3.1., GraphPad, La Jolla, CA, USA) software with an alpha level set to *p* < 0.05. Data were checked for normality prior to analysis and outliers greater than 2 standard deviations from the mean were excluded. First, unpaired t-tests were conducted on behavioral dependent variables to establish an effect of PSS exposure on anxiety- and anhedonia-like behavior. A double-median split of time spent in the OA of the EPM and total sucrose consumed during SPT was used to phenotype rats as stress-Susceptible, Intermediate, or Resilient. Next, one-way analyses of variance (ANOVAs) were used to compare behavioral data between phenotypes (Control, Susceptible, and Resilient), as well as brain monoamine and metabolite levels and turnover. DA and 5-HT turnover were calculated according to Slotkin et al. [47]: DA turnover = (HVA + DOPAC)/DA; 5-HT turnover = (5-HIAA)/(5-HT). Tukey’s multiple comparison tests were used following significant main effects. Spearman correlations were used to assess linear relationships between brain measurements and behavior. Data are expressed as mean ± standard error of mean (SEM). 

## 3. Results

### 3.1. Effect of PSS on Behavior 

To determine if TMT altered anxiety-like behavior overall, t-tests were used to compare dependent variables between TMT and Control groups. TMT-exposed rats spent less time in the open arms of the EPM [t(52) = 2.072, *p* = 0.043] and displayed increased mean startle responses [t(45) = 2.168, *p* = 0.0355; one control and five TMT rats were removed due to equipment malfunction, one Control rat was removed for exhibiting startle responses greater than 2 standard deviations away from the mean] compared to Controls. TMT-exposed rats also showed lower sucrose preference [t(52) = 2.517, *p* = 0.0149] and decreased sucrose consumption (mL) [t (52) = 3.111, *p* = 0.003] relative to Controls. 

Rats were next classified as Susceptible, Intermediate, or Resilient according to a double-median split of the time spent in the open arms of the EPM (median = 73.7 s) and sucrose intake (median = 67.25 mL) during sucrose preference testing. Rats were classified as Resilient (*n* = 12) if they spent more than the median time spent in open arms and consumed more sucrose than the median. Rats were classified as Susceptible (*n* = 11) if they spent less than the median time in the open arms of EPM and consumed less sucrose than the median. Rats that spent a lower amount of time than the median in the open arms of EPM and consumed more sucrose than the median, or that spent a higher amount of time than the median in the open arms of EPM and consumed a lower amount of sucrose than the median, were classified as Intermediate. Rats in the Intermediate phenotype were excluded from further analyses, as the present study aims to study behavioral extremes in response to stress. Of the Control rats that were exposed to the same context but without TMT and subsequently tested in the EPM and ASR, only two met the criteria for susceptibility, indicating that this phenotype is not pre-existing and instead is induced by PSS. Following phenotyping, a subset of rats (Control *n* = 8, Susceptible *n* = 8, Resilient *n* = 8) representing the same effect of phenotype as the larger group were selected for use in the present study. However, brain processing for two Susceptible rats rendered the data unusable (Susceptible *n* = 6). Behavioral data from the entire cohort (*n* = 54) is found in our prior publication [32]. Behavioral results from the subset of rats selected for use in the present tissue punch analysis are described here and in Table 1. 

A one-way ANOVA revealed a significant effect of Phenotype on time spent in the open arms of the EPM [F (2,19) = 9.372, *p* = 0.002] with Susceptible rats showing less time spent in the OA relative to Control (*p* = 0.001) and Resilient (*p* = 0.016) rats. There was also a significant effect of Phenotype on time spent in the closed arms [F (2,19) = 6.164, *p* = 0.009] with Susceptible rats spending more time in the CA compared to Controls (*p* = 0.006). CA entries and OA entries did not differ between phenotypes. 

Due to ASR equipment malfunction, data files from two Resilient rats and one Susceptible rat were corrupted and could not be included in analyses. A one-way ANOVA revealed no differences in percent habituation between phenotypes, but a significant difference in mean startle amplitude [F (2,16) = 20.70, *p* < 0.001]. Susceptible rats have greater mean startle amplitude than both Control (*p* < 0.001) and Resilient (*p* < 0.001) rats.

No phenotypic differences were detected in sucrose preference; however, there was an effect on sucrose consumed [F (2,19) = 17.74, *p* < 0.001]. Susceptible rats decreased sucrose intake compared to Control (*p* < 0.001) and Resilient (*p* < 0.001) rats. 

A one-way ANOVA revealed significant effects of Phenotype on time spent in the dark chamber of the L-D box task [F (2,19) = 3.64, *p* = 0.046] with no significant multiple comparisons. No significant phenotypic differences were detected in latencies to the light or dark sides of the chamber. 

During TMT exposure, rats did not display freezing behavior, therefore no effect of Phenotype on time spent freezing was observed. There were no significant effects of Phenotype on freezing during context re-exposure. Two Control rats were removed from context re-exposure analysis due to corrupted video files. 

One-way ANOVAs compared dependent variables between different phases of the estrous cycle. The estrous cycle phase at the time of TMT exposure had no effect on later anxiety-like behavior or sucrose intake. The estrous cycle at the time of EPM/ASR testing also had no influence on anxiety-like behavior. See our prior publication for more details [32]. 

### 3.2. Effect of PSS on Brain Norepinephrine 

**Figure 2:**
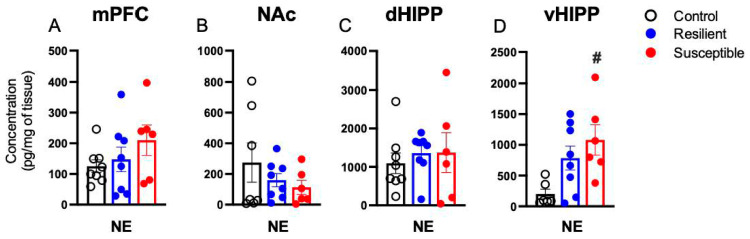
Effect of predator scent stress on brain levels of norepinephrine (NE) in the (**A**) medial prefrontal cortex (mPFC), (**B**) nucleus accumbens (NAc), (**C**) dorsal hippocampus (dHIPP), and (**D**) ventral hippocampus (vHIPP). vHIPP NE was increased in Susceptible females compared to Controls (**D**). Values expressed as mean ± SEM. # = *p* < 0.05 compared to Control.

Tissue collection from the vHIPP of one Control rat was processed incorrectly and not analyzed. One NE value from a Control rat value was identified as a statistical outlier (>2 standard deviations from the mean) and removed from vHIPP NE analysis. Brain NE levels in the mPFC, NAc, and dHIPP did not differ by phenotype (Figure 2A–C). A main effect of Phenotype on vHIPP NE levels was observed [F (2,17) = 4.972, *p* = 0.02; Figure 2D]. Susceptible rats showed elevated NE levels compared to Controls (*p* = 0.017). There was a negative correlation between vHIPP NE and sucrose consumed (r = −0.442, *p* = 0.045).

### 3.3. Effect of PSS on Brain Dopamine, Metabolites, and Dopamine Turnover 

**Figure 3:**
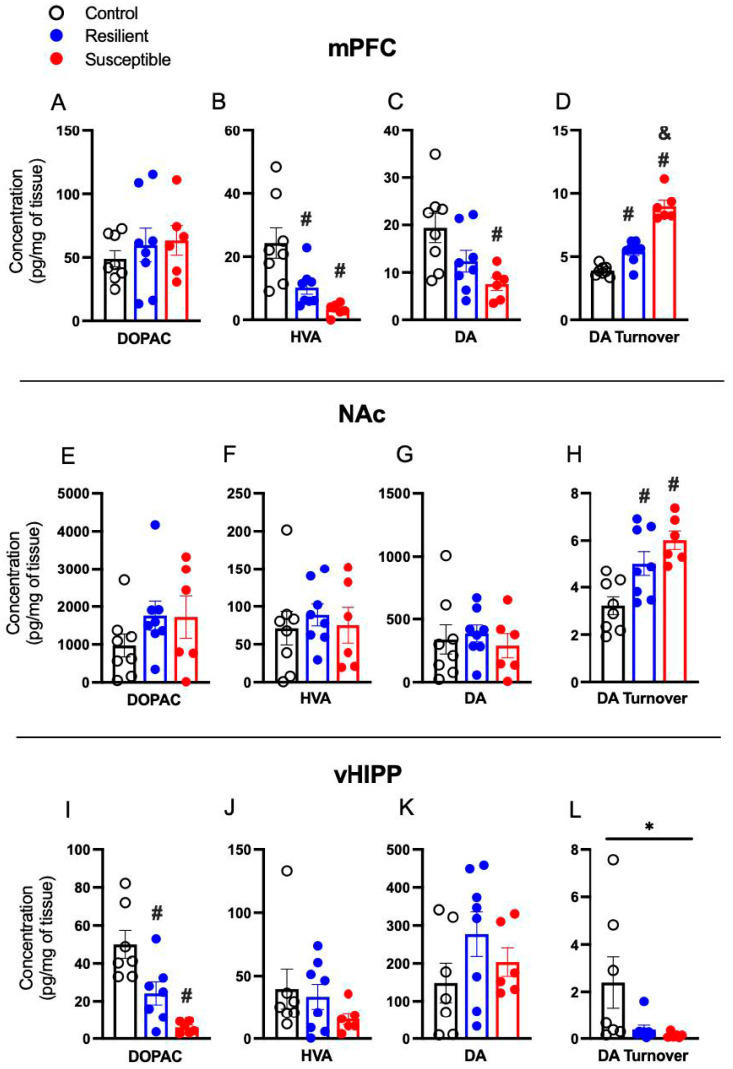
Effect of predator scent stress on brain levels of dopamine, dopamine metabolites, and dopamine turnover in the medial prefrontal cortex (mPFC), nucleus accumbens (NAc), and ventral hippocampus (vHIPP). (**A**) There was no effect of Phenotype on mPFC DOPAC. (**B**) mPFC HVA was decreased in Susceptible and Resilient rats compared to Control. (**C**) mPFC DA was decreased in Susceptible compared to Control. (**D**) DA turnover was increased in Susceptible and Resilient rats compared to Controls, and Susceptible show greater DA turnover compared to Resilient. No effect of Phenotype was observed in NAc DOPAC, HVA, or DA (**E**–**G**). (**H**) Susceptible and Resilient showed increased NAc DA turnover compared to Control. (**I**) vHIPP DOPAC was decreased in Susceptible and Resilient rats compared to Control. No effect of Phenotype was observed in vHIPP HVA (**J**) or DA (**K**). (**L**) Significant mean differences were detected in vHIPP DA turnover. Values expressed as mean ± SEM. * = *p* < 0.05 effect of Phenotype, # = *p* < 0.05 compared to Control, & = *p* < 0.05 compared to Resilient.

In the mPFC, no phenotype differences in mPFC DOPAC were detected (Figure 3A). There was a significant effect of Phenotype on the DA metabolite HVA [F (2,19) = 10.03, *p* = 0.001] and multiple comparisons revealed both Susceptible (*p* = 0.001) and Resilient (*p* = 0.02) rats decreased HVA relative to Controls (Figure 3B). mPFC DA differed by phenotype [F (2,19) = 5.386, *p* = 0.01], with decreased DA in Susceptible rats compared to Controls (*p* = 0.01; Figure 2C). There was a significant effect of phenotype on DA turnover [F (2,19) = 65.20, *p* = 0 < 0.001] with both Susceptible (*p* < 0.001) and Resilient (*p* = 0.01) rats showing increased DA turnover compared to Controls, and Susceptible showing a greater increase than Resilient (*p* < 0.001; Figure 3D).

In the NAc, there was no effect of Phenotype on DOPAC, HVA, or DA (Figure 3E–G). However, a significant effect of Phenotype was detected for DA turnover [F (2,19) = 9.876, *p* = 0.001] with both Susceptible (*p* = 0.001) and Resilient (*p* = 0.02) displaying increased DA turnover relative to Controls (Figure 3H).

There were no significant effects of Phenotype on DOPAC, HVA, DA, or DA turnover in the dHIPP (not shown). In the vHIPP, there was a significant effect of Phenotype in DOPAC [F (2,17) = 14.15, *p* < 0.001] with decreases in both Susceptible (*p* < 0.001) and Resilient (*p* = 0.01) compared to Controls (Figure 3I). No significant differences were detected in vHIPP HVA and DA (Figure 3J,K). There was an effect of Phenotype on vHIPP DA turnover [F (2,17) = 3.715, *p* = 0.04], with no significant multiple comparisons (Figure 3L). 

DA and DA turnover correlated with several behaviors. mPFC DA was negatively correlated with time spent in the dark chamber of the L-D box (r = −0.429, *p* = 0.046). Time spent in the OA of the EPM was negatively correlated with DA turnover in the mPFC (r = −0.620, *p* = 0.002) and NAc (r = −0.5622, *p* = 0.007). Sucrose consumed was negatively correlated with DA turnover in the mPFC (r = −0.811, *p* < 0.001) and NAc (r = −0.5828, *p* = 0.004). DA turnover in the NAc was positively correlated with time spent in the dark chamber of the L-D box (r = 0.462, *p* = 0.031).

### 3.4. Effect of PSS on Brain Serotonin, 5-Hydroxyindoleacetic Acid, and Serotonin Turnover 

**Figure 4:**
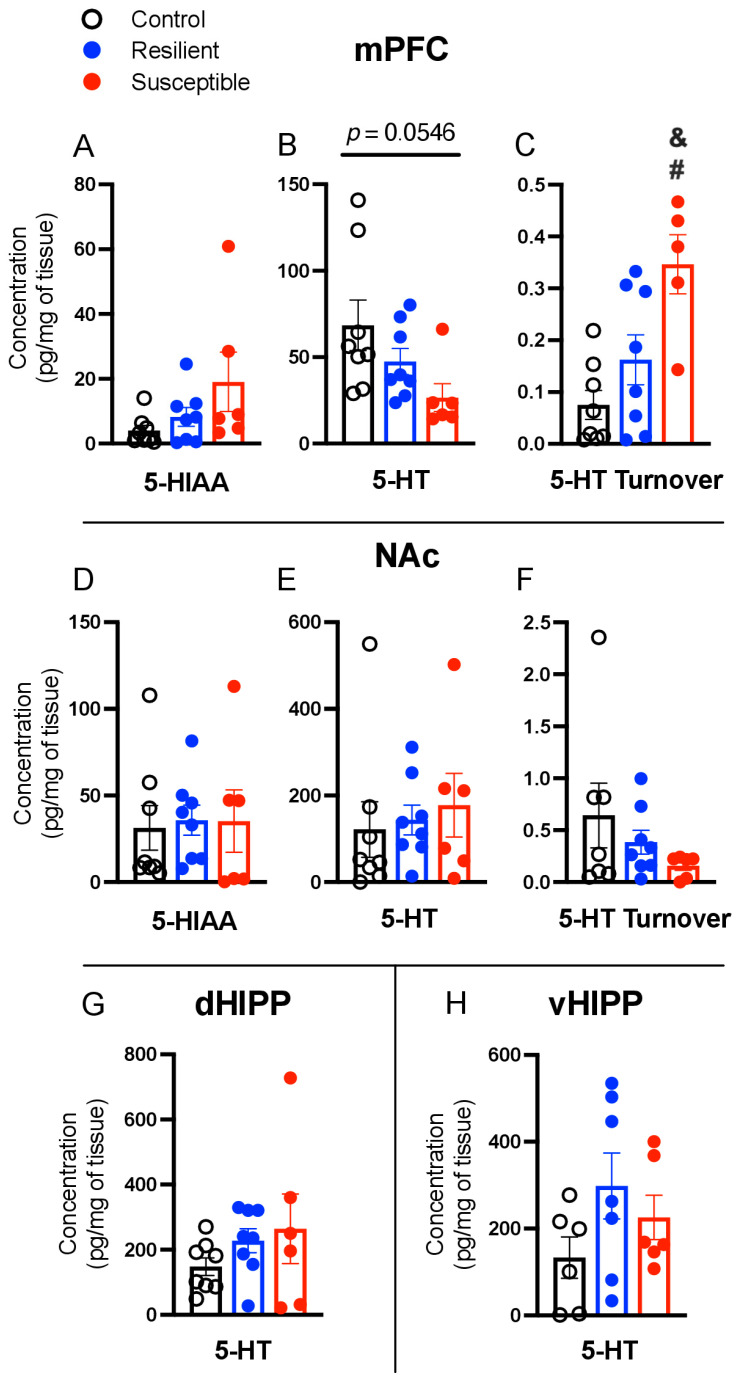
Effect of predator scent stress on brain levels of serotonin (5-HT), 5-hydroxyindoleacetic acid (5-HIAA), and 5-HT turnover in the medial prefrontal cortex (mPFC), nucleus accumbens (NAc), and dorsal (dHIPP) and ventral hippocampus (vHIPP). In the mPFC, (**A**) no effect on 5-HIAA was detected, (**B**) there was a non-significant trend (*p* = 0.056) in 5-HT, and (**C**) a significant increase in 5-HT turnover for stress-Susceptible rats compared to Resilient and Control. No effect of stress was observed in the NAc (**D**–**F**), dHipp (**G**), or vHIPP (**H**). Values expressed as mean ± SEM. # = *p* < 0.05 compared to Control, & = *p* < 0.05 compared to Resilient.

No significant differences were detected in 5-HIAA levels in the mPFC (Figure 4A) There was a trend for an effect of Phenotype on 5-HT [F (2,19) = 3.360, *p* = 0.056; Figure 4B] and a significant difference in 5-HT turnover [F (2,19) = 8.617, *p* = 0.002; Figure 4C]. There was greater 5-HT turnover in Susceptible compared to Control (*p* = 0.002) and Resilient (*p* = 0.03) rats, with no difference between Resilient and Control. No phenotype effects were found in 5-HIAA, 5-HT, or 5-HT turnover in the NAc, dHIPP, or vHIPP (Figure 4D–H). A significant positive correlation between mPFC 5-HT turnover and ASR mean startle (*p* = 0.014, r = 0.554) was detected.

## 4. Discussion

The present study examined the effect of a single exposure to PSS on tissue levels of monoamines, their metabolites, and turnover in the mPFC, NAc, dHIPP, and vHIPP of female rats. Heterogenous responses to PSS allowed for the separation of stress-exposed female rats into Susceptible and Resilient phenotypes. Phenotypic patterns of behavior in EPM, sucrose preference, and the light–dark box were accompanied by distinct monoamine changes. Stress-susceptibility was accompanied by reduced DA and HVA and a trend for reduced 5-HT in the mPFC. Susceptible rats showed an elevated turnover of DA and 5-HT within the mPFC and elevation of DA turnover in the NAc. Susceptible rats also exhibited increased vHIPP NE and decreased DOPAC, and an effect of phenotype was observed in vHIPP DA turnover. Though brain monoamine and metabolites were assessed days to weeks after behavioral tasks, decreased mPFC DA and greater DA turnover in the mPFC and NAc were correlated with increased anxiety-like behavior and anhedonia. 

### 4.1. Norepinephrine

Susceptible female rats exhibited increased NE concentrations in the vHIPP four weeks after PSS, in agreement with results in male rats one day after repeated predator and psychosocial stress [28]. As described in Table 2, most studies of brain monoamines report no changes in hippocampal NE hours to weeks after stress exposure [26,29,31] with one study finding decreased hippocampal NE three days after footshock [48]. Of note, and given their functional differences [49], we analyzed dorsal and ventral hippocampal regions separately, finding no differences in NE levels in the dHIPP; many prior studies analyzed the hippocampus as a whole, which complicates comparisons across studies. 

NE plays a key role in the stress response and memory consolidation and is implicated in PTSD. Years to decades after traumatic exposure, male combat veterans diagnosed with PTSD show increased CSF NE levels that positively correlate with symptom severity as identified by the Clinician-Administered PTSD scale [14]. The perception of a stressor activates the locus coeruleus (LC), which sends strong noradrenergic projections to the hippocampus that are thought to reinforce the storage of long-term memories [50]. NE hyperactivity, specifically through the LC-to-HIPP projections, is hypothesized as a potential mechanism for how some PTSD symptoms develop [51,52]. Although we assessed the anxiety- and anhedonia-like behaviors induced by PSS weeks prior to brain analysis, decreased sucrose consumption during the SPT was accompanied by greater vHIPP NE concentrations, indicating a relationship between increased hippocampal NE weeks after stress exposure and greater anhedonia. 

As seen in Table 2, De La Garza and Mahoney [25], Hayley et al. [26], Wilson et al. [28], and Muneoka et al. [31] find that prior stress exposure increases mPFC NE concentrations in male rats regardless of stress-susceptibility or resilience, when assessed mere hours to 5 days after stress. Sixteen days after repeated predator scent stress, Tseilikman et al. [29] found that elevated mPFC NE is associated with resilience in males. Here we found no effect of phenotype or PSS on mPFC NE concentrations 4 weeks after stress, potentially due to the longer period of time between stress exposure and NE assessment. However, Isingrini et al. [30] found that 26 h after repeated social defeat stress, male mice phenotyped as Susceptible or Resilient based on social avoidance parameters also show no changes in mPFC NE concentrations, in agreement with the present results. Collectively, these findings indicate that sex, the type of stressor, and the length of time between stress and assessment may all influence mPFC NE concentrations. 

### 4.2. Dopamine

We found that Susceptible females exhibit decreased mPFC DA concentrations, in agreement with some studies in males [25] but not others that report increases [27,28] or no change in mPFC DA [24,26,29,31]. DA turnover in the mPFC is clearly associated with stress exposure and susceptibility in males when assessed immediately following stress [25], 24 h after stress [28], and 16 days after stress [29] (see Table 2). Here we found that 4 weeks after PSS, Susceptible females also show elevated mPFC DA turnover, an effect mediated by low DA and HVA in Susceptible females that is accompanied by no differences in DOPAC between phenotypes. 

It is well-established that the DA system is activated in response to acute and chronic stressors [39,53,54] and involves a complex metabolism. After release, DA is recycled through reuptake or degradation. DA accumulated in the cytosol is metabolized by monoamine oxidase (MAO), to DOPAL, which is converted by aldehyde dehydrogenase to DOPAC. After reuptake to glial cells, DA converted to DOPAC is then further metabolized by COMT to HVA [55]. COMT is highly expressed in the mPFC [56]. Given the metabolism of DA, decreased HVA but not DOPAC in Susceptible rats suggests reduced COMT availability in the mPFC in Susceptible female rats. This hypothesis is in agreement with clinical findings showing that the COMT Val^158^Met polymorphism is associated with lowered enzyme activity and increased susceptibility to develop PTSD after stress exposure in humans [57]. 

Elevated DA turnover in Susceptible females was also observed in the NAc. However, studies in males find no difference in NAc DA turnover following stress exposure 2 to 5 days after [27,31]. Elevated DA turnover in the NAc of males has been found in the conditioned fear (footshock) model when assessed immediately after stress (see Table 2). In the present study, greater increases in DA turnover in both the NAc and mPFC were associated with greater anxiety-like behavior and anhedonia. Clinical data support our DA turnover findings related to anhedonia: Patients diagnosed with depressive illnesses exhibit decreased HVA and elevated DA turnover that positively correlates with symptom severity [58]. Further, women with PTSD report greater anhedonia than men [59,60].

Though no phenotypic effects on DA or its metabolites were observed in the dHIPP, Resilient and Susceptible rats showed decreased vHIPP DOPAC compared to Controls and there was an effect of phenotype on DA turnover. Hippocampal monoamines are dysregulated after repeated PSS exposure in high- versus low-anxiety male rats. As seen in Table 2, Tseilikman et al. [29] found that high-anxiety rats exhibit decreased DA and increased DOPAC and DA turnover 16 days after PSS, and Muneoka et al. [31] show male rats that develop learned helplessness after footshock exhibit elevated DA turnover 5 days later. These differences could be attributed to the different stress models or in the amount of time between stress exposure tissue analyses between our study and those that previously assessed vHIPP DA metabolism. Additionally, sex may also play a role. 

### 4.3. Serotonin

We found a trend toward phenotypic differences in mPFC 5-HT, with Controls displaying greater concentrations. The type of stress model has clear effects on mPFC 5-HT concentrations in male rats (see Table 2). Models employing repeated stress and a forced swim find susceptibility is associated with decreased mPFC 5-HT immediately after [25], 24 h after [28], or 16 days after stress [29]. This effect is not observed 5 days after footshock [31] or 2 days after isolation stress [27], which results in no effect and increased mPFC 5-HT, respectively. In female Susceptible rats, we find elevated 5-HT turnover in the mPFC 4 weeks after PSS relative to Resilient and Control rats. This is consistent with work performed in male rodents where greater 5-HT turnover is found in Susceptible rats immediately after forced swim stress [25]. However, no effects on 5-HT turnover were found in Susceptible rats 5 days after footshock [31], and decreased 5-HT turnover was found 16 days after repeated predator scent stress [29]. Thus, findings regarding mPFC 5-HT turnover are not consistent across models and are also potentially influenced by sex and time since stress.

Serotonergic projections to the PFC primarily originate in the dorsal raphe nucleus and blunted mPFC 5-HT is noted in the WKY (stress-Susceptible) rat strain [61]. It is possible that a single exposure to PSS in females blunts 5-HT release in Susceptible rats. Phenotype-specific 5-HT system alterations also could be a result of decreased 5-HT synthesis. Wistar rats exposed to repeated predator stress show a reduction in mPFC tryptophan hydroxylase expression, important for the 5-HT synthesis [28]. Altered 5-HT may also be pre-existing. Clinical research shows brain serotonin turnover is elevated in unmedicated patients with major depressive disorder (MDD) compared to healthy controls. Subsequent SSRI treatment reduced 5-HT turnover accompanied by decreased MDD symptoms [62]. We observed no significant differences in 5-HT in any other brain regions four weeks after PSS, in contrast to previous studies 24 h after predator stress [28], 5 days after footshock [31], and 2 days after isolation stress in males [27]. It is likely these differences are due to differences in strain, sex, timing, or a result of a single exposure compared to repeated exposures and warrant further investigation.

### 4.4. Limitations

In drawing conclusions from these data, we acknowledge limitations in our design. The use of homogenized samples does not permit the determination of whether the analyte levels originate presynaptically or extracellularly. Tissue was collected at a single time point and thus did not reflect the dynamic changes in monoamine levels after a brief episode of re-exposure to the PSS context. Further, we segregated rats into Susceptible and Resilient phenotypes and eliminated rats classified as “Intermediate” (behavioral measures falling between the two phenotypes). Future studies should examine dependent variables in the entire population of TMT-exposed rats to examine relationships between behavior and brain measures along a continuous scale. 

The lack of direct male comparisons in this study limits our ability to make definitive conclusions about the effect of sex on monoamine regulation; however, there are known baseline sex differences in monoamine turnover across brain regions. Females display lower DA turnover in the vmPFC, NAc, and dHIPP and lower 5-HT turnover in the vmPFC compared to males [63]. Further, while we established that the estrous phase during TMT exposure and subsequent behavioral measures do not influence phenotype [32], the estrous phase was not determined on the day of tissue collection in the present study. mPFC and HIPP 5-HT turnover is lower in proestrus than diestrus [64], which may have influenced our observations of increased 5-HT turnover in the mPFC and/or the null effects observed in the HIPP. In future studies, the examination of estrous cycle effects on monoamine function should be prioritized. 

## 5. Conclusions

Analysis of brain monoamines after stress has previously been limited to male rodents. Using a single predator scent stress exposure rat model of PTSD, we identified phenotype-specific alterations in monoamines, their metabolites, and turnover rates in female rats related to anxiety-like behavior and anhedonia and consistent with clinical research. The most pronounced findings between phenotypes were observed in the mPFC, NAc, and vHIPP, finding susceptibility to stress was accompanied by reduced DA and HVA in the mPFC, increased DA turnover in the mPFC and NAc, and increased 5-HT turnover in the mPFC. Moreover, Susceptible rats exhibited elevated NE in the vHIPP. DA turnover in multiple brain regions was associated with susceptibility in females and was correlated with more severe anxiety-like (EPM and L-D box) behavior and anhedonia (sucrose consumption), suggesting regulation of dopamine turnover may be a contributing factor in stress-susceptibility in females.

## Figures and Tables

**Table 1:** Results of one-way ANOVAs for behavioral tests between Control, Resilient, and Susceptible. Mean ± SEM; bold = *p* < 0.05 effect of Phenotype, underline = *p* < 0.05 v. Control v. Susceptible, * = *p* < 0.05 Resilient v. Susceptible.

	Control (*n* = 8) ^+^	Resilient (*n* = 8)	Susceptible (*n* = 6)
Exposure freezing (s)	0 ± 0	0 ± 0	0 ± 0
Re-exposure freezing (s)	7.83 ± 14.06	18.50 ± 52.33	4 ± 7.27
**EPM: time in OA (s)**	104.7 ± 14.08	88.01 ± 2.59 *	43.49 ± 8.15 *
**EPM: time in CA (s)**	114.5 ± 6.81	138.9 ± 12.34	175.1 ± 16.63
EPM: OA entries (#)	20.88 ± 5.12	17.38 ± 2.81	11.50 ± 3.37
EPM: CA entries (#)	25.25 ± 3.08	34.63 ± 3.95	34.76 ± 7.90
**Sucrose consumed (mL)**	82.13 ± 3.46	75.00 ± 1.77 *	53.33 ± 5.00 *
Sucrose preference (%)	89.64 ± 2.43	87.86 ± 2.38	82.58 ± 4.49
**L-D: time in dark (s)**	216.3 ± 35.15	298.4 ± 13.30	315 ± 31.17
**L-D: time in light (s)**	383.8 ± 35.15	301.6 ± 31.17	285.0 ± 31.17
L-D: latency to dark (s)	6.286 ± 2.41	13.38 ± 3.85	16.00 ± 6.29
L-D: latency to light (s)	24.75 ± 7.48	23.00 ± 6.36	27.00 ± 8.05

^+^ For re-exposure comparisons, Control (*n* = 6). EPM = elevated plus maze, OA = open arms, CA = closed arms, L-D = light–dark box.

**Table 2:** Brain monoamine levels and turnover in rodents with a history of stress. mPFC = medial prefrontal cortex, NAc = nucleus accumbens, HIPP = hippocampus, NE = norepinephrine, DA = dopamine, 5-HT = serotonin.

	Reference	Stress Model	Strain	Sex	NE	DA	DA Turnover	5-HT	5-HT Turnover
**mPFC**	Morrow et al., 2000 [24]	Footshock	Sprague-Dawley rats	Male		↔ stressed v. CTRL	↑ stressed v. CTRL		
	Muneoka et al., 2020 [31]	Footshock	Sprague-Dawley	Male	↔ Susceptible v. CTRL & Resilient	↔ Susceptible v. CTRL & Resilient	↔ Susceptible v. CTRL & Resilient	↔ Susceptible v. CTRL & Resilient	↔ Susceptible v. CTRL & Resilient
	De La Garza & Mahoney, 2004 [25]	Forced swim	Wistar and WKY rats	Male	↑ stressed v. CTRL ↔ Susceptible v. Resilient	↓ Susceptible v. CTRL & Resilient	↑ Susceptible v. CTRL & Resilient	↓ Susceptible v. CTRL & Resilient	↑ Susceptible v. CTRL & Resilient
	Isingrini et al., 2016 [30]	Social defeat	C57BL/6 mice	Male	↔ Susceptible v. CTRL & Resilient				
	Hayley et al., 2001 [26]	Predator scent	C57BL/6ByJ & BALB/cByJ mice	Male	↑ stressed v. CTRL	↔ stressed v. CTRL		↔ stressed v. CTRL	
	Han et al., 2011 [27]	Post-weaning isolation	Sprague-Dawley rats	Male		↑ stressed v. CTRL	↔ stressed v. CTRL	↑ stressed v. CTRL	↔ stressed v. CTRL
	Wilson et al., 2014 [28]	Predator scent + psychosocial stress	Sprague-Dawley rats	Male	↑ stressed v. CTRL	↑ stressed v. CTRL		↓ stressed v. CTRL	
	Tseilikman et al., 2020 [29]	Repeated predator scent	Wistar rats	Male	↔ Susceptible v. CTRL ↑ Resilient v. CTRL	↔ Susceptible & Resilient v. CTRL	↑ Susceptible v. CTRL ↔ Resilient v. Susceptible & CTRL	↓ Susceptible v. CTRL ↔ Resilient v. Susceptible & CTRL	↓ Susceptible v. CTRL ↔ Resilient v. Susceptible & CTRL
	Morrow et al., 2000 [24]	Acute and repeated predator scent	Sprague-Dawley rats	Male		↔ stressed v. CTRL	↑ stressed v. CTRL		
	**Present ** **Results**	**Predator scent**	**Sprague-Dawley**	**Female**	**↔** **Susceptible v. CTRL & Resilient**	**↓** **Susceptible v. CTRL**	**↑ ** **Susceptible v. CTRL & Resilient** **↑ ** **Resilient v. CTRL**	**↔** **Susceptible v. CTRL & Resilient**	**↑ ** **Susceptible v. CTRL & Resilient**
**NAc**	Morrow et al., 2000 [24]	Footshock	Sprague-Dawley rats	Male		↔ stressed v. CTRL	↑ stressed v. CTRL		
	Muneoka et al., 2020 [31]	Footshock	Sprague-Dawley	Male	↔ Susceptible v. CTRL & Resilient	↔ Susceptible v. CTRL & Resilient	↔ Susceptible v. CTRL & Resilient	↓ Susceptible v. Resilient	↔ Susceptible v. CTRL & Resilient
	Isingrini et al., 2016 [30]	Social defeat	C57BL/6 mice	Male	↔ Susceptible v. CTRL & Resilient				
	Han et al., 2011 [27]	Post-weaning isolation	Sprague-Dawley rats	Male		↑ stressed v. CTRL	↔ stressed v. CTRL	↑ stressed v. CTRL	↑ stressed v. CTRL
	Morrow et al., 2000 [24]	Predator scent	Sprague-Dawley rats	Male		↔ stressed v. CTRL	↔ stressed v. CTRL		
	**Present ** **Results**	**Predator scent**	**Sprague-Dawley**	**Female**	**↔** **Susceptible v. CTRL & Resilient**	**↔** **Susceptible v. CTRL & Resilient**	**↑ ** **Susceptible v. CTRL** **↑ ** **Resilient v. CTRL**	**↔** **Susceptible v. CTRL & Resilient**	**↔** **Susceptible v. CTRL & Resilient**
**HIPP**	Sziray et al., 2007 [49]	Footshock	Sprague-Dawley	Male	↓ stressed v. CTRL			↓ stressed v. CTRL	
	Muneoka et al., 2020 [31]	Footshock	Sprague-Dawley	Male	↔ Susceptible v. CTRL & Resilient	↔ Susceptible v. CTRL & Resilient	↑ Susceptible v. CTRL & Resilient	↔ Susceptible v. CTRL & Resilient	↔ Susceptible v. CTRL & Resilient
	Hayley et al., 2001 [26]	Predator scent	C57BL/6ByJ & BALB/cByJ mice	Male	↔ stressed v. CTRL	↔ stressed v. CTRL		↔ stressed v. CTRL	
	Wilson et al., 2014 [28]	Repeated PSS + psychosocial stress	Sprague-Dawley	Male	↑ stressed v. CTRL	↔ stressed v. CTRL		↓ stressed v. CTRL	
	Tseilikman et al., 2020 [29]	Repeated predator scent	Wistar	Male	↔ Susceptible v. CTRL & Resilient	↓ Susceptible v. CTRL & Resilient	↑ Susceptible v. CTRL & Resilient	↓ Susceptible v. CTRL & Resilient	↑ Susceptible v. CTRL & Resilient
	**Present ** **Results** **(vHIPP)**	**Predator scent**	**Sprague-Dawley**	**Female**	**↑ ** **Susceptible v. CTRL** **↔** **Resilient v. CTRL & Susceptible**	**↔** **Susceptible v. CTRL & Resilient**	**↔** **Susceptible v. CTRL & Resilient**	**↔** **Susceptible v. CTRL & Resilient**	**↔** **Susceptible v. CTRL & Resilient**

^“^high anxiety” groups or more stress-Susceptible strains are depicted as “Susceptible” in the table above. Bold indicates present study results in females for comparison.

## Data Availability

Not applicable.
